# Predicting Covid-19 infection and death rates among E.U. minority populations in the absence of racially disaggregated data through the use of US data comparisons

**DOI:** 10.1093/eurpub/ckad164

**Published:** 2023-09-15

**Authors:** Paola Cecchi Dimeglio, Robert E Fullilove, Catherine Cecchi, Yann Cabon, Jessica Rosenberg

**Affiliations:** Harvard Law School (Center on the Legal Profession) and Harvard Kennedy School (Women and Public Policy), Harvard University, Cambridge, MA, USA; Department of Sociomedical Sciences and Health Policy and Management, Mailman School of Public Health, Columbia University, New York, NY, USA; Société Francaise de Sante Publique and Société Francophone Santé Environnement, Montpellier, France; Behavioral Insights Institute, Cambridge, MA, USA; Department of Epidemiology, Mailman School of Public Health, Columbia University, New York, NY, USA

## Abstract

**Background:**

The E.U.’s lack of racially disaggregated data impedes the formulation of effective interventions, and crises such as Covid-19 may continue to impact minorities more severely. Our predictive model offers insight into the disparate ways in which Covid-19 has likely impacted E.U. minorities and allows for the inference of differences in Covid-19 infection and death rates between E.U. minority and non-minority populations.

**Methods:**

Data covering Covid-19, social determinants of health and minority status were included from 1 March 2020 to 28 February 2021. A systematic comparison of US and E.U. states enabled the projection of Covid-19 infection and death rates for minorities and non-minorities in E.U. states.

**Results:**

The model predicted Covid-19 infection rates with 95–100% accuracy for 23 out of 28 E.U. states. Projections for Covid-19 infection and mortality rates among E.U. minority groups illustrate parallel trends to US rates.

**Conclusions:**

Disparities in Covid-19 infection and death rates by minority status likely exist in patterns similar to those observed in US data. *Policy Implications*: Collecting data by race/ethnicity in the E.U. would help document health disparities and craft more targeted health interventions and mitigation strategies.

## Introduction

Crises highlight conditions and spur innovation. Since the World Health Organization declared SARS-CoV-2 (Covid-19) a pandemic on 11 March 2020, it has challenged the global public health system in numerous ways, some of which are difficult to quantify. Covid-19 has strained healthcare staff, facilities, systems, resources, tools and processes. It has shown what can occur when data gathering is limited or restricted.^1^ In many places, analyses and the formation of predictive models remain challenging. In many settings, the impact of Covid-19 on minority communities has been difficult to determine.^2–4^

In order to confront public health crises such as Covid-19-, through short-, medium- and long-term management approaches, the collection and analysis of disaggregated data is essential. US and E.U. data collection processes for infection and mortality rates have differed dramatically, due largely to E.U. policies governing racial and ethnic data collection.^5^

Collecting disaggregated data by race and ethnicity is commonplace in the USA. Such data allow for the swift identification of disparities, as seen during the early months of the Covid-19 pandemic in the USA.^6^ In fact, disaggregated data quickly illuminated that certain racial and ethnic minority groups had a higher risk of contracting and dying from Covid-19 than their counterparts.^7^ It is difficult to form successful public responses that are not informed by this type of data.

The E.U.’s policies regarding racial and ethnic data collection poses such a difficulty.^8^ Since World War II, collecting racial and ethnic data has been prohibited in housing, employment and health. The resulting data gap means an absence of the knowledge necessary to facilitate tailored public health strategies for vaccine dissemination that allow for targeted outreach in disproportionately impacted communities. With a few exceptions, there is a paucity of Covid-19 infection and mortality data available in the E.U. when compared with the USA in terms of gender and race.^9^

In the USA, disparities in health and the quality of healthcare are well-documented. US communities of color have historically experienced higher mortality from infectious diseases. Covid-19 is no exception and has exacerbated these disparities.

Given the socioeconomic drivers known in the USA, similar disparities likely exist among ethnic and racial minorities in the E.U. Despite critical differences between the USA and the E.U.—including factors for mitigating health disparities, such as more robust safety net services in the E.U.—disparities are still likely to persist throughout the E.U.^10^

In public health, effective management and mitigation rely on data. Without means of gauging differential health outcomes for European minority communities, formulating adequate, targeted public health interventions remains difficult, and public health crises such as Covid-19 are likely to continue to more severely impact minority communities.^11^

The research and the predictive model we have developed, using the social determinants of health (SDOH), provides a solution to the E.U. data gap. This article aims to provide a novel approach to modeling the ways in which the Covid-19 pandemic has likely disproportionately impacted minority communities in the E.U. by leveraging the USA as a comparison.^12^ In the absence of racial and ethnic data, our predictive model successfully bridges the evidence gap and provides new avenues whereby public health authorities may design effective interventions for these communities in the E.U.

## Methods

### Composition of the dataset

The period of analysis included data collected in the E.U. and the USA from 1 March 2020 to 28 February 2021 (https://www.ecdc.europa.eu/en/covid-19/data). The UK is excluded from the dataset because they are no longer considered a member of the E.U. (https://www.cdc.gov/coronavirus/2019-ncov/covid-data/covid-19-data-sources.html). For both the E.U. and the USA, data covering outside territories such as Corsica, France, are included in the analysis. In addition, the District of Columbia is counted as a separate state in the USA due to the granularity of available data. Key categories including gross domestic product (GDP), SDOH and Covid-19 infection and death rates were used to conduct a comparison between the USA and the E.U.

The dataset is organized around three key informational themes: Covid-19, SDOH and minority status (racial/ethnic). Covid-19 infection and death rates were extracted from the European Centre for Disease Prevention and Control (See Supplementary File; predictive model) for the E.U. and from the Center for Disease Control and Prevention (CDC) [important to note that the UK evidence could have been discussed, as the NHS shares similarities with other European health systems (https://www.ncbi.nlm.nih.gov/pmc/articles/PMC8087292/?report=reader)] and the Johns Hopkins Coronavirus Resource Center for the USA (https://coronavirus.jhu.edu/map.html). The US data contained information about gender, race/ethnicity and age, whereas the E.U. data only included information about gender and age (the infection and death rate are age not-standardized).

In order to estimate the proportion of the population potentially categorized as minorities in the E.U., information was derived from secondary sources including the World Directory of Minorities and Indigenous Peoples and the United Nations High Commissioner for Refugees (World Directory of Minorities and Indigenous Peoples). These data were triangulated with information from other publicly available sources such as l’Observatoire des inégalités (for France) (https://www.inegalites.fr). Minorities are defined as those whose race/ethnicity is other than White [including the following: African-American/Black, Alaska Native/American Indian, Asian, Hispanic/Latinx, Multiracial (those who identify with two or more of the aforementioned races/ethnicities) and Native Hawaiian/Pacific Islander], individuals that identify as LGBTQ+, individuals with disabilities as recognized by US law under the Americans with Disability Act (ADA) and any other category falling under the ADA or recognized by the E.U. Directive on Disability.

SDOH data including information about the economy, labor (employment), education, politics, environment, housing, medical care, government, public health, psychology and behavior were derived from the Organization for Economic Co-Operation and Development (OECD), CDC, US Census Bureau, EuroStat and Air Quality Data (https://aqicn.org/data-platform/covid19/).

## Results

**Figure 1:**
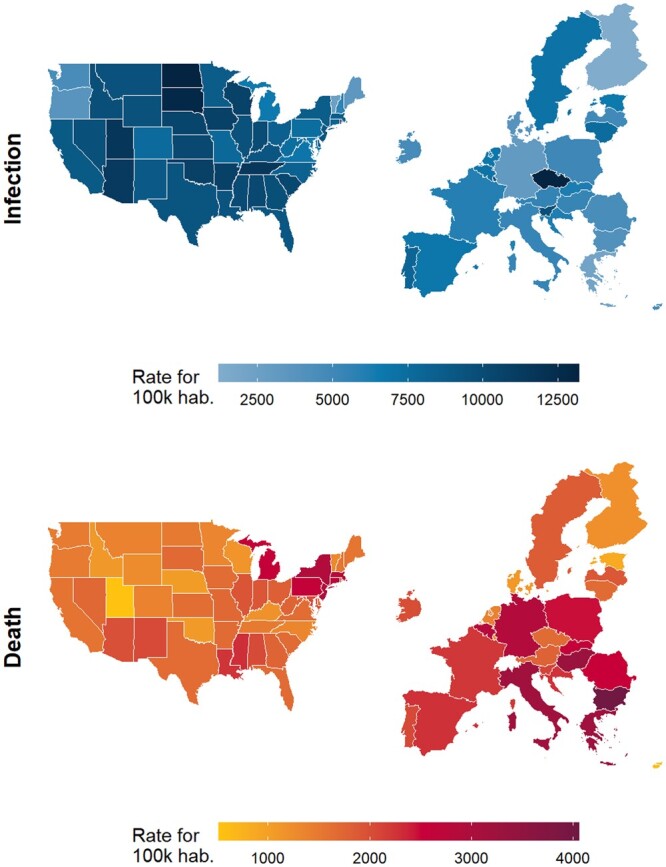
Observed Covid-19 infection and death rates in the USA and E.U.

Figure 1 displays the Covid-19 infection and death rates per 100 000 residents in the USA and in the E.U. (total population). Six range categories for infection rate per 100 000 residents are included: (1) less than 2500, (2) from 2501 to 5000, (3) from 5001 to 7500, (4) from 7501 to 10 000, (5) from 10 001 to 12 500 and (6) above 12 501. Five range categories for death rate per 100 000 residents are included: (1) less than 1000, (2) from 1001 to 2000, (3) from 2001 to 3000, (4) from 3001 to 4000 and (5) above 4001.

Supplementary table S1 reports Covid-19 rates of infection and rates of death for corresponding E.U. states and US states. Six ranges present infection and death rates from less than 2500 per 100k people and less than 1000 per 100k people, respectively, to infection rates up to and surpassing 12 500 per 100k people and mortality rates exceeding 4001 per 100k people. E.U. and US states are grouped according to these rates.

The following is observed for the comparison between Covid-19 infection rates in the USA and the E.U.: in the USA, 12 states fall in the first three range categories (Range 1: HI; Range 2: ME, OR, VT, WA; Range 3: DC, MD, MI, NH, PA, VA, WV). The majority of the US states fall in the fourth and fifth range categories (Range 4: AK, CA, CO, CT, DE, FL, GA, ID, IL, IN, KY, LA, MA, MN, MS, MO, MT, NV, NJ, NM, NY, NC, OH, TX, WY; Range 5: AL, AZ, AR, IA, KS, NE, OK, RI, SC, TN, UT, WI). Only ND and SD fall in the sixth range category.

In the E.U., most of the states fall in the first three range categories (Range 1: Finland, Greece; Range 2: Bulgaria, Cyprus, Denmark, Germany, Ireland, Latvia and Romania; Range 3: Austria, Belgium, Croatia, Estonia, France, Hungary, Italy, Malta, Netherlands, Poland, Slovakia, Spain, Sweden). Four of the E.U. states fall in the fourth range (Lithuania, Luxembourg, Portugal, Slovenia). No E.U. states fall in the fifth range, however, the Czech Republic is in the sixth range.

The following is observed for the comparison between Covid-19 death rates in the USA and the E.U.: in the USA, all of the states fall in the first three range categories (Range 1: AK, UT; Range 2: AZ, AR, CA, CO, DE, FL, GA, HI, ID, IL, IN, IA, KS, KY, ME, MN, MO, MT, NE, NV, NH, NC, ND, OH, OK, OR, RI, SC, SD, TN, TX, VT, VA, WA, WV, WI, WY; Range 3: AL, CT, DC, LA, MD, MA, MI, MS, NJ, NM, NY, PA). No US states fall in range categories 4, 5 or 6.

In the E.U., similar trends are observed in that all of the E.U. states fall in the first four range categories (Range 1: Cyprus, Estonia, Iceland, Norway; Range 2: Austria, Czech Republic, Denmark, Finland, Ireland, Latvia, Lithuania, Luxembourg, Malta, Netherlands, Sweden; Range 3: Belgium, Croatia, France, Germany, Liechtenstein, Poland, Portugal, Romania, Slovenia, Spain; Range 4: Greece, Hungary, Italy). No E.U. states fall in range categories 5 or 6.

**Figure 2:**
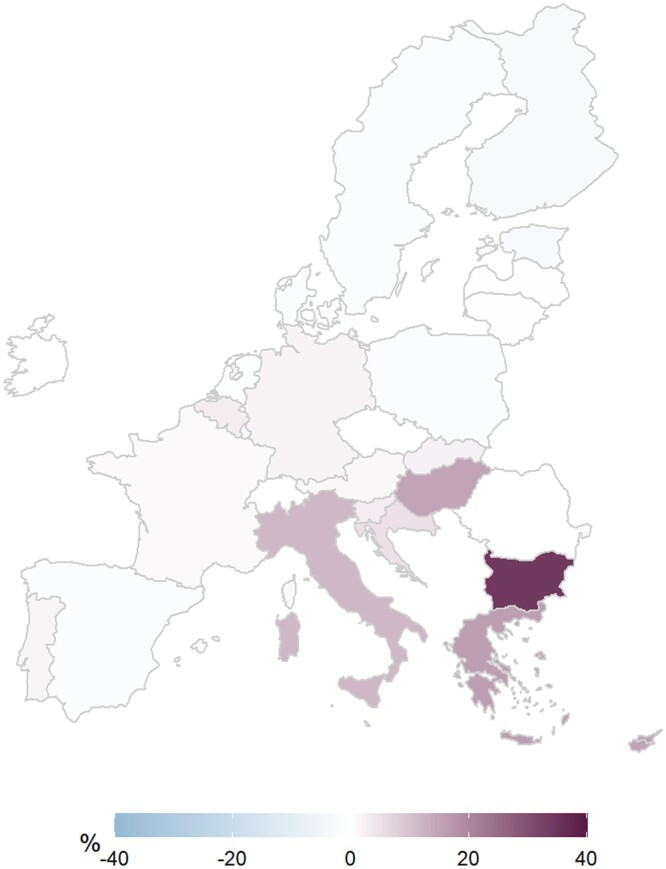
Difference in Covid-19 infection rate percentiles between the predictive model and the data reported by E.U. states

Figure 2 presents the difference in Covid-19 infection rate percentiles between the predictive model and the data reported by E.U. states. The percentile difference is composed of five range categories: (1): lower than 15.1% (2): from −15% to −5.1%; (3) from −5% to 5%; (4) from 5.1% to 15% and (5) above 15.1%.

Supplementary table S2 reports the accuracy of the predictive model. It shows the repartition of E.U. states in Covid-19 infection rate percentiles between the predictive model and reported data.

As observed in Supplementary table S2, no states fall into range categories 1 and 2. Most of the E.U. states fall into range category 3: Austria, Belgium, Croatia, Czech Republic, Denmark, Estonia, Finland, France, Germany, Ireland, Latvia, Lithuania, Luxembourg, Malta, Netherlands, Poland, Portugal, Romania, Slovakia, Slovenia, Spain and Sweden. The two states with a percentile difference in range 4 are Hungary and Italy. Bulgaria, Cyprus and Greece are in the fifth range.

**Figure 3:**
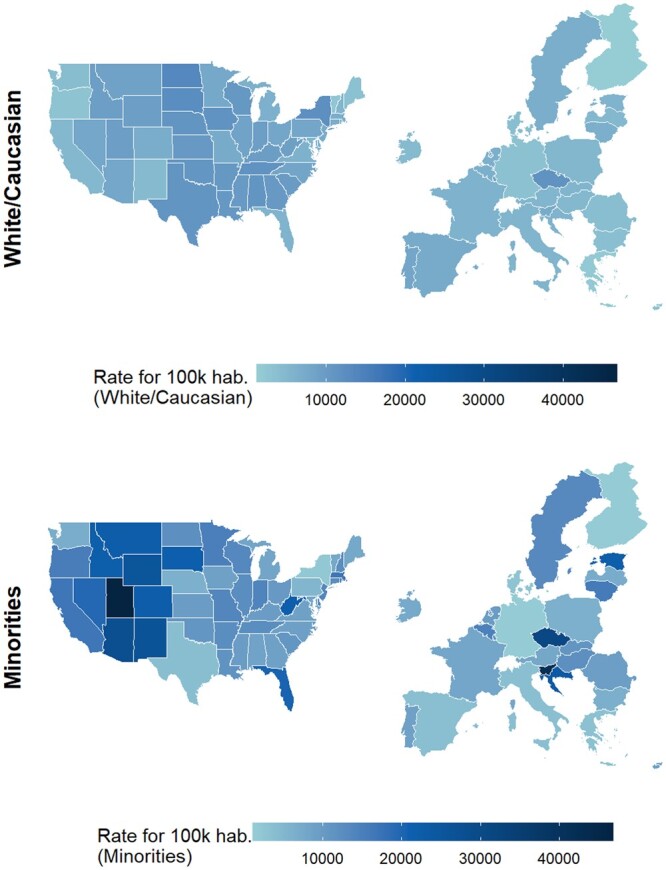
Predictive model projection Covid-19 infection rate by race (White/Caucasian and Minorities) in the E.U based on US data

Figure 3 displays a projection of Covid-19 infection rate by race (white vs. minorities) in the E.U. based on the predictive model. Five range categories of rate per 100 000 residents are included: (1) below 10 000, (2) from 10 001 to 20 000, (3) from 20 001 to 30 000, (4) from 30 001 to 40 000 and (5) above 40 001.

Supplementary table S3 shows a projection of Covid-19 infection rate by race (white vs. minorities) in the E.U. based on the predictive model.

For the comparison of the Covid-19 infection rate between the USA and the E.U., Supplementary table S3 shows the following for the White population: in the USA, the vast majority of the states fall in range 1 (AK, AZ, CA, CO, CT, DC, DE, FL, HI, ID, IL, IN, KY, MA, MD, ME, MI, MN, MO, MT, NC, NH, NJ, NM, NV, OH, OR, PA, RI, SC, UT, VA, VT, WA, WV, WY) and the remaining states fall in range 2 (AL, AR, GA, IA, KS, LA, MS, ND, NE, NY, OK, SD, TN, TX, WI). Not US states fall into ranges 3, 4 or 5.

Similar trends are predicted in the E.U. where most of the states fall in range 1 (Austria, Belgium, Bulgaria, Croatia, Cyprus, Denmark, Estonia, Finland, France, Germany, Greece, Hungary, Ireland, Italy, Latvia, Lithuania, Luxembourg, Malta, Netherlands, Poland, Portugal, Romania, Slovakia, Slovenia, Spain, Sweden) and only the Czech Republic falls in range 2.

For the comparison of the Covid-19 infection rate between the USA and the E.U., the following is observed for the minorities population: in the USA, most of the states fall in range 1 (AL, DC, GA, HI, IA, KS, KY, MD, ME, MI, MS, NC, NE, NY, PA, TX, VA, VT, WA), in range 2 (AK, AR, CA, CT, DE, IL, IN, LA, MA, MN, MO, ND, NH, NJ, NV, OH, OK, OR, SC, TN, WI) and in range 3 (AZ, CO, FL, ID, MT, NM, RI, SD, WV, WY). No US states fall in range 4 and only UT falls in range 5.

In the E.U., most states fall in range 1 (Austria, Bulgaria, Cyprus, Denmark, Finland, France, Germany, Greece, Ireland, Italy, Latvia, Netherlands, Poland, Portugal, Romania, Spain) and in range 2 (Belgium, Hungary, Lithuania, Luxembourg, Malta, Slovakia, Sweden). The remaining states fall in range 3 (Croatia, Estonia), range 4 (Czech Republic) and range 5 (Slovenia).

**Figure 4:**
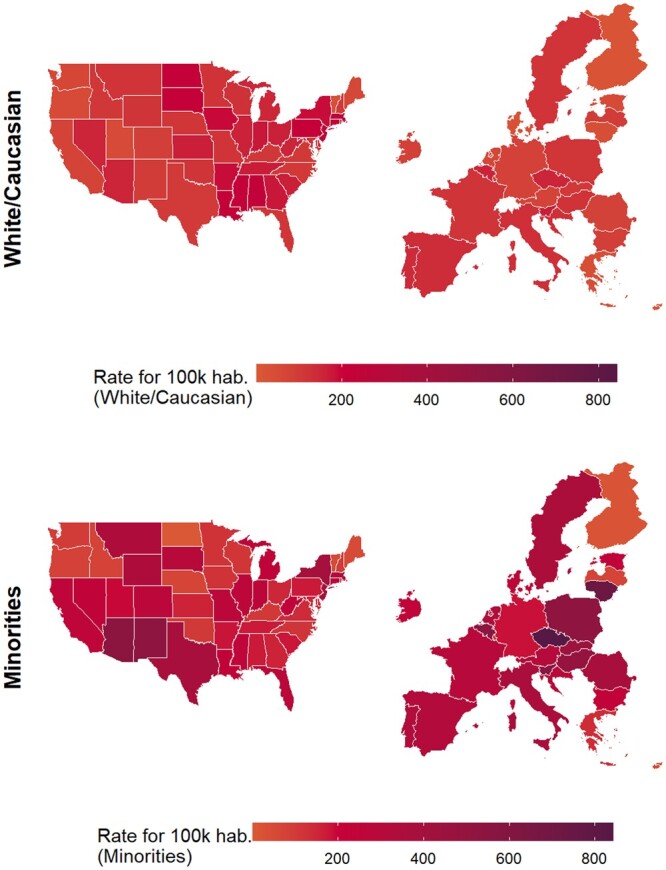
Predictive model projection Covid-19 death rate by race (White/Caucasian and Minorities) in the E.U based on US data

Figure 4 illustrates the projection of Covid-19 death rate by race (White vs. minorities) in the E.U. based on the predictive model. Four range categories of rate per 100 000 residents are included (1) below 200; (2) from 201 to 400; (3) from 401 to 600 and (4) above 601.

Supplementary table S4 shows a projection of Covid-19 death rate by race (white vs. minorities) in the E.U. based on the predictive model.

For the comparison of the Covid-19 death rate between the USA and the E.U., the following is observed for the White population: in the USA, the vast majority of the states are in range 1 (AK, AZ, AR, CA, CO, CT, DE, DC, FL, GA, HI, ID, IL, IN, IA, KS, KY, ME, MD, MI, MN, MO, MT, NE, NV, NH, NM, NY, NC, OH, OK, OR, SC, TN, TX, UT, VT, VA, WA, WV, WI, WY) and only a few states are in range 2 (AL, LA, MA, MS, ND, NJ, PA, RI, SD). No US states fall in ranges 3 or 4.

Similar trends are predicted in the E.U., where all of the states are in range 1 (Austria, Belgium, Bulgaria, Croatia, Cyprus, Czech Republic, Denmark, Estonia, Finland, France, Germany, Greece, Hungary, Ireland, Italy, Latvia, Lithuania, Luxembourg, Malta, Netherlands, Poland, Portugal, Romania, SC, Slovakia, Slovenia, Spain, Sweden). No E.U. states fall in ranges 2, 3 or 4.

For the comparison of the Covid-19 death rate between the USA and the E.U., the following is observed for the minorities population: in the USA, the majority of the states are in range 1 (AK, AL, AR, DE, GA, HI, IA, ID, KS, KY, MD, ME, MN, NC, ND, NE, NH, OH, OK, OR, PA, RI, SC, TN, UT, VA, VT, WA, WI) and range 2 (CA, CO, CT, DC, FL, IL, IN, LA, MA, MI, MO, MS, MT, NJ, NV, NY, SD, TX, WV, WY). Two states fall into range 3 (AZ, NM). No US states fall into range 4.

In the E.U., the majority of the states fall in ranges 1 (Cyprus, Finland, Germany, Greece, Latvia, Luxembourg) and 2 (Austria, Bulgaria, Croatia, Denmark, Estonia, France, Ireland, Italy, Netherlands, Portugal, Romania, Spain, Sweden). The remaining states fall in range 3 (Belgium, Hungary, Malta, Poland, Slovakia, Slovenia) and range 4 (Czech Republic, Lithuania).

## Discussion

The Covid-19 pandemic has caused worldwide devastation for communities, health systems and nations, and is likely to have long-term adverse implications.^13^ Despite successes in vaccination development and dissemination, the emergence of Covid-19 variants poses new challenges. Governments must respond effectively. Future public health goals, pandemic research priorities and policy implementation must consider the SDOH and persistent disparities.

Disparities in health and the quality of healthcare experienced by minorities are a well-documented element of persistent ‘racial inequalities’, especially in the USA. Race can influence where individuals live, creating differential access to health resources.^14^ Drivers include access to quality education, employment, income and wealth, housing, living in a clean environment and access to fresh fruits and vegetables, public housing and environmental toxins and so on that can have adverse health outcomes.^15^^,^^16^ In 2021, the uninsured rate in the USA was higher for certain groups. This disparity in insurance coverage may be a contributing factor to the disparities in Covid-19 infection and death rates in the country. These factors disproportionately impact communities of color and make mitigation strategies such as physical distancing more difficult.

Since race is not a biologically active variable, racial disparities must be analyzed within their socioeconomic framework in order to understand their root causes.^17^^,^^18^ The predictive model presented here adopts a reliable means for bridging the data gap and as such the reduction of health disparities in the E.U and vice versa. It accurately anticipated current rates of infection and mortality by race in the USA and E.U. and has potential as a forecasting tool applicable to vaccine dissemination and the spread of the Covid-19 delta variant in the E.U. for White Caucasian/minority populations.

Research combining quantitative and qualitative data is critical in contextualizing the experiences of minority populations in data.^19^^,^^20^ Accurate, consistent and culturally relevant information can be made readily available to all communities. Effective interventions and strategies can be tailored to fit local circumstances, facilitate timely linkage to care, reduce disease transmission, curtail disease incidence and ultimately diminish health disparities.^21^

The Covid-19 pandemic provides opportunities for innovation and for synergies with human rights and sustainable development goals and agendas prioritized by the United Nations. Efforts must be intensified to mitigate and remove barriers faced by racial and ethnic minorities, indigenous peoples, refugees, migrants, people with disabilities and those struggling to access safe and affordable healthcare.^22^

Our research bolsters this argument, demonstrating that patterns of Covid-19 infection and mortality in the USA are parallel to projections of such rates in the E.U., indicating differential impacts on minority communities. The absence of identifying data has long posed a barrier to the pursuit of equitable development. The E.U. has struggled to create and implement targeted Covid-19 intervention strategies and vaccination efforts. In the absence of such data, the application of the predictive model can enable policymakers and practitioners to predict patterns of infection and mortality among minority communities.

## Supplementary Material

ckad164_Supplementary_Data

## Data Availability

We are committed to ensuring the transparency and reproducibility of our research. All relevant data, including datasets, codes and Supplementary Materials, are publicly available. Key points The lack of racially disaggregated data in the European Union hinders the development of effective interventions to address health disparities. Informed public policies that incorporate racially disaggregated data show better outcomes in terms of prevention and response strategies, especially in times of crisis. Minorities in the European Union may continue to experience more severe impacts from crises such as Covid-19, including higher infection and death rates. Our predictive models have demonstrated high accuracy in forecasting Covid-19 infection and death rates with 95–100% accuracy for 23 out of 28 E.U. states. Similar to the USA, it is likely that disparities in Covid-19 infection and death rates based on minority status exist in the European Union, highlighting the need for race/ethnicity data collection to identify and address health disparities effectively.
