# Supplementary material for: Predicting Covid-19 infection and death rates among E.U. minority populations in the absence of racially disaggregated data through the use of US data comparisons
Source: Eur J Public Health. 2023 Sep 15;34(1):176–80. doi: 10.1093/eurpub/ckad164 (PMC10843944; doi:10.1093/eurpub/ckad164)
Supplement: ckad164_Supplementary_Data [file ckad164_supplementary_data.zip › ckad164_Supplementary_Data/ejph-2023-05-om-0249-File006.docx]

**Supplementary Table 1: Observed Covid-19 infection and death rates in the U.S. and the E.U.**

| **Range of Infection Rate per 100k residents** | **Region** | **Country or US State name (alphabetical order)** | **Range of Death Rate per 100k residents** | **Region** | **Country or US State name (alphabetical order)** |
| --- | --- | --- | --- | --- | --- |
| Below 2,500 | E.U. | Finland; Greece | Below 1,000 | E.U. | Cyprus; Estonia |
|  | U.S. | HI |  | U.S. | AK; UT |
| From 2,501 to 5,000 | E.U. | Bulgaria; Cyprus; Denmark; Germany; Ireland; Latvia; Romania | From 1,001 to 2,000 | E.U. | Austria; Czech Republic; Denmark; Finland; Ireland; Latvia; Lithuania; Luxembourg; Malta; Netherlands; Sweden |
|  | U.S. | ME; OR; VT; WA |  | U.S. | AR; AZ; CA; CO; DE; FL; GA; HI; IA; ID; IL; IN; KS; KY; ME; MN; MO; MT; NC; ND; NE; NH; NV; OH; OK; OR; RI; SC; SD; TN; TX; VA; VT; WA; WI; WV; WY |
| From 5,001 to 7,500 | E.U. | Austria; Belgium; Croatia; Estonia; France; Hungary; Italy; Malta; Netherlands; Poland; Slovakia; Spain; Sweden | From 2,001 to 3,000 | E.U. | Belgium; Croatia; France; Germany; Poland; Portugal; Romania; Slovakia; Slovenia; Spain |
|  | U.S. | DC; MD; MI; NH; PA; VA; WV |  | U.S. | AL; CT; DC; LA; MA; MD; MI; MS; NJ; NM; NY; PA |
| From 7,501 to 10,000 | E.U. | Lithuania; Luxembourg; Portugal; Slovenia | From 3,001 to 4,000 | E.U. | Greece; Hungary; Italy |
|  | U.S. | AK; CA; CO; CT; DE; FL; GA; ID; IL; IN; KY; LA; MA; MN; MO; MS; MT; NC; NJ; NM; NV; NY; OH; TX; WY |  | U.S. |  |
| From 10,001 to 12,500 | E.U. |  | Above 4,001 | E.U. | Bulgaria |
|  | U.S. | AL; AR; AZ; IA; KS; NE; OK; RI; SC; TN; UT; WI |  | U.S. |  |
| Above 12,501 | E.U. | Czech Republic |  |  |  |
|  | U.S. | ND; SD |  |  |  |
| Note: U.S. states displayed in alphabetic order: Alaska (AK); Alabama (AL); Arkansas (AR); Arizona (AZ); California (CA); Colorado (CO); Connecticut (CT); Dist. Of Columbia (DC); Delaware (DE); Florida (FL); Georgia (GA); Hawaii (HI); Iowa (IA); Idaho (ID); Illinois (IL); Indiana (IN); Kansas (KS); Kentucky (KY); Louisiana (LA); Massachusetts (MA); Maryland (MD); Maine (ME); Michigan (MI); Minnesota (MN); Missouri (MO); Mississippi (MS); Montana (MT); North Carolina (NC); North Dakota (ND); Nebraska (NE); New Hampshire (NH); New Jersey (NJ); New Mexico (NM); Nevada (NV); New York (NY); Ohio (OH); Oklahoma (OK); Oregon (OR); Pennsylvania (PA); Rhode Island (RI); South Carolina (SC); South Dakota (SD); Tennessee (TN); Texas (TX); Utah (UT); Virginia (VA); Vermont (VT); Washington (WA); Wisconsin (WI); West Virginia (WV); Wyoming (WY) | | | | | |
