# Supplementary material for: Predicting Covid-19 infection and death rates among E.U. minority populations in the absence of racially disaggregated data through the use of US data comparisons
Source: Eur J Public Health. 2023 Sep 15;34(1):176–80. doi: 10.1093/eurpub/ckad164 (PMC10843944; doi:10.1093/eurpub/ckad164)
Supplement: ckad164_Supplementary_Data [file ckad164_supplementary_data.zip › ckad164_Supplementary_Data/ejph-2023-05-om-0249-File007.docx]

**Supplementary Table 2: Difference between the predictive model & reported data**

| **Range difference** | **E.U. countries** (**alphabetical order)** |
| --- | --- |
| Less than -15% |  |
| From -15% to -5% |  |
| From -5% to 5% | Austria; Belgium; Croatia; Czech Republic; Denmark; Estonia; Finland; France; Germany; Ireland; Latvia; Lithuania; Luxembourg; Malta; Netherlands; Poland; Portugal; Romania; Slovakia; Slovenia; Spain; Sweden |
| From 5% to 15% | Hungary; Italy |
| More than 15% | Bulgaria; Cyprus; Greece |
