# Supplementary material for: Predicting Covid-19 infection and death rates among E.U. minority populations in the absence of racially disaggregated data through the use of US data comparisons
Source: Eur J Public Health. 2023 Sep 15;34(1):176–80. doi: 10.1093/eurpub/ckad164 (PMC10843944; doi:10.1093/eurpub/ckad164)
Supplement: ckad164_Supplementary_Data [file ckad164_supplementary_data.zip › ckad164_Supplementary_Data/ejph-2023-05-om-0249-File008.docx]

**Supplementary Table 3: Predictive model projected Covid-19 infection rate (White/Caucasian & Minorities) in the U.S. and the E.U.**

| **Infection Rate per 100k residents (White/Caucasian)** | **Region** | **Country or US State name (alphabetical order)** | **Infection Rate per 100k residents (Minorities)** | **Region** | **Country or US State name (alphabetical order)** |
| --- | --- | --- | --- | --- | --- |
| Below 10,000 | E.U. | Austria; Belgium; Bulgaria; Croatia; Cyprus; Denmark; Estonia; Finland; France; Germany; Greece; Hungary; Ireland; Italy; Latvia; Lithuania; Luxembourg; Malta; Netherlands; Poland; Portugal; Romania; Slovakia; Slovenia; Spain; Sweden | Below 10,000 | E.U. | Austria; Bulgaria; Cyprus; Denmark; Finland; France; Germany; Greece; Ireland; Italy; Latvia; Netherlands; Poland; Portugal; Romania; Spain |
|  | U.S. | AK; AZ; CA; CO; CT; DC; DE; FL; HI; ID; IL; IN; KY; MA; MD; ME; MI; MN; MO; MT; NC; NH; NJ; NM; NV; OH; OR; PA; RI; SC; UT; VA; VT; WA; WV; WY |  | U.S. | AL; DC;GA; HI; IA; KS; KY; MD; ME; MI; MS; NC; NE; NY; PA; TX; VA; VT; WA |
| From 10,001 to 20,000 | E.U. | Czech Republic | From 10,001 to 20,000 | E.U. | Belgium; Hungary; Lithuania; Luxembourg; Malta; Slovakia; Sweden |
|  | U.S. | AL; AR; GA; IA; KS; LA; MS; ND; NE; NY; OK; SD; TN; TX; WI |  | U.S. | AK; AR; CA; CT; DE; IL; IN; LA; MA; MN; MO; ND; NH; NJ; NV; OH; OK; OR; SC; TN; WI |
| From 20,001 to 30,000 | E.U. |  | From 20,001 to 30,000 | E.U. | Croatia; Estonia |
|  | U.S. |  |  | U.S. | AZ; CO; FL; ID; MT; NM; RI; SD; WV; WY |
| From 30,001 to 40,000 | E.U. |  | From 30,001 to 40,000 | E.U. | Czech Republic |
|  | U.S. |  |  | U.S. |  |
| Above 40,001 | E.U. |  | Above 40,001 | E.U. | Slovenia |
|  | U.S. |  |  | U.S. | UT |
| Note: U.S. states displayed in alphabetic order: Alaska (AK); Alabama (AL); Arkansas (AR); Arizona (AZ); California (CA); Colorado (CO); Connecticut (CT); Dist. Of Columbia (DC); Delaware (DE); Florida (FL); Georgia (GA); Hawaii (HI); Iowa (IA); Idaho (ID); Illinois (IL); Indiana (IN); Kansas (KS); Kentucky (KY); Louisiana (LA); Massachusetts (MA); Maryland (MD); Maine (ME); Michigan (MI); Minnesota (MN); Missouri (MO); Mississippi (MS); Montana (MT); North Carolina (NC); North Dakota (ND); Nebraska (NE); New Hampshire (NH); New Jersey (NJ); New Mexico (NM); Nevada (NV); New York (NY); Ohio (OH); Oklahoma (OK); Oregon (OR); Pennsylvania (PA); Rhode Island (RI); South Carolina (SC); South Dakota (SD); Tennessee (TN); Texas (TX); Utah (UT); Virginia (VA); Vermont (VT); Washington (WA); Wisconsin (WI); West Virginia (WV); Wyoming (WY) | | | | | |
