# Supplementary material for: Predicting Covid-19 infection and death rates among E.U. minority populations in the absence of racially disaggregated data through the use of US data comparisons
Source: Eur J Public Health. 2023 Sep 15;34(1):176–80. doi: 10.1093/eurpub/ckad164 (PMC10843944; doi:10.1093/eurpub/ckad164)
Supplement: ckad164_Supplementary_Data [file ckad164_supplementary_data.zip › ckad164_Supplementary_Data/ejph-2023-05-om-0249-File009.docx]

**Supplementary Table 4: Predictive model projected Covid-19 death rate (White/Caucasian & Minorities) in the U.S. and the E.U.**

| **Death Rate per 100k residents (White/Caucasian)** | **Region** | **Country or US State name (alphabetical order)** | **Death Rate per 100k residents (Minorities)** | **Region** | **Country or US State name (alphabetical order)** |
| --- | --- | --- | --- | --- | --- |
| Below 200 | E.U. | Austria; Belgium; Bulgaria; Croatia; Cyprus; Czech Republic; Denmark; Estonia; Finland; France; Germany; Greece; Hungary; Ireland; Italy; Latvia; Lithuania; Luxembourg; Malta; Netherlands; Poland; Portugal; Romania; Slovakia; Slovenia; Spain; Sweden | Below 200 | E.U. | Cyprus; Finland; Germany; Greece; Latvia; Luxembourg |
|  | U.S. | AK; AR; AZ; CA; CO; CT; DC; DE; FL; GA; HI; IA; ID; IL; IN; KS; KY; MD; ME; MI; MN; MO; MT; NC; NE; NH; NM; NV; NY; OH; OK; OR; SC; TN; TX; UT; VA; VT; WA; WI; WV; WY |  | U.S. | AK; AL; AR; DE; GA; HI; IA; ID; KS; KY; MD; ME; MN; NC; ND; NE; NH; OH; OK; OR; PA; RI; SC; TN; UT; VA; VT; WA; WI |
| From 201 to 400 | E.U. |  | From 201 to 400 | E.U. | Austria; Bulgaria; Croatia; Denmark; Estonia; France; Ireland; Italy; Netherlands; Portugal; Romania; Spain; Sweden |
|  | U.S. | AL; LA; MA; MS; ND; NJ; PA; RI; SD |  | U.S. | CA; CO; CT; DC; FL; IL; IN; LA; MA; MI; MO; MS; MT; NJ; NV; NY; SD; TX; WV; WY |
| From 401 to 600 | E.U. |  | From 401 to 600 | E.U. | Belgium; Hungary; Malta; Poland; Slovakia; Slovenia |
|  | U.S. |  |  | U.S. | AZ; NM |
| Above 601 | E.U. |  | Above 601 | E.U. | Czech Republic; Lithuania |
|  | U.S. |  |  | U.S. |  |
| Note: U.S. states displayed in alphabetic order: Alaska (AK); Alabama (AL); Arkansas (AR); Arizona (AZ); California (CA); Colorado (CO); Connecticut (CT); Dist. Of Columbia (DC); Delaware (DE); Florida (FL); Georgia (GA); Hawaii (HI); Iowa (IA); Idaho (ID); Illinois (IL); Indiana (IN); Kansas (KS); Kentucky (KY); Louisiana (LA); Massachusetts (MA); Maryland (MD); Maine (ME); Michigan (MI); Minnesota (MN); Missouri (MO); Mississippi (MS); Montana (MT); North Carolina (NC); North Dakota (ND); Nebraska (NE); New Hampshire (NH); New Jersey (NJ); New Mexico (NM); Nevada (NV); New York (NY); Ohio (OH); Oklahoma (OK); Oregon (OR); Pennsylvania (PA); Rhode Island (RI); South Carolina (SC); South Dakota (SD); Tennessee (TN); Texas (TX); Utah (UT); Virginia (VA); Vermont (VT); Washington (WA); Wisconsin (WI); West Virginia (WV); Wyoming (WY) | | | | | |
