# Supplementary material for: Predicting Covid-19 infection and death rates among E.U. minority populations in the absence of racially disaggregated data through the use of US data comparisons
Source: Eur J Public Health. 2023 Sep 15;34(1):176–80. doi: 10.1093/eurpub/ckad164 (PMC10843944; doi:10.1093/eurpub/ckad164)
Supplement: ckad164_Supplementary_Data [file ckad164_supplementary_data.zip › ckad164_Supplementary_Data/ejph-2023-05-om-0249-File010.docx]

**Supplementary file**

**Method section**

**Predictive Model**

We took an important step in creating the predictive model: we developed a matching system between states in the U.S. and nations in the E.U., since no established scientific consensus existed for their comparison.

The predictive model utilized patterns and attributes from the database to make predictions and extract rules for different elements. Specifically, the model used Covid-19 infection and death rates per 100,000 residents in the U.S. and E.U. to predict the rates projected by race (white Caucasian / minorities) in the E.U. states.

The construction of the predictive model involved four main steps.

The first step was to compare the total number of infections and the death rates observed in the U.S. with the E.U. over time (Figure 1).

Secondly, U.S. states were matched to E.U. states. In our model, $E$ represents the E.U. states and $\mathcal{S}$ represents the combination of the U.S. states. Each E.U. nation and U.S. state were compared among a number of common variables defined by $X$ combined by a weight $w.$The $X$ variables used to establish the best match between the U.S. and the E.U states were disposable income per capita (USD), employment rate, (per capita (PC)), homicide rate (per 100,000 individuals), life expectancy at birth (year), number of rooms per person (ratio), perceived social network support (PC), perception of corruption (PC),  self-evaluation of life satisfaction (per capita), share of households with internet broadband access (PC), share of labor force with at least secondary education (PC), standardized mortality rate (per 1,000 individuals), unemployment rate (PC), voter turnout in general election (PC), GDP ((2018, 2019, 2020, 2021) in USD (PC)), and percentile of Covid-19 infection rate and death rate (population total and per race/minority).

$\left\| X\left( E \right),X(s) \right\|$ represented the Euclidean distance (i.e., straight line). It is mathematically defined as the distance between points A and B, which is based on the premise that every instance in the dataset can be represented as a point in N-dimensional space and the distance between any two points can be optimized. The Euclidean distance can be used as an effective measure in comparative studies.

Thus, $S_{E}$ represented the best association between  $E$ and $\mathcal{S}$ as follows:

$$S_{E}=argmin_{s\in\mathcal{S}}\left( \left\| X\left( E \right),X(s) \right\|_{w} \right)$$

Then, $d_{E}$represented the estimated total number of deaths in the E.U states based on the previous equation.  The function $f$ was used to project the number of deaths in the E.U. states based on their combination of corresponding U.S. states, as:

$$d_{E}=f\left( S_{E} \right)$$

The matching score corresponded to the quadratic difference between the number of COVID-19 death observed ($D_{E}$) in the E.U. states and those estimated by the model $d_{E}.$Each E.U. state had a weight^1^ defined by  $p (between 1-4 )$. These weights were assigned based on the length of time that a state has been in the E.U. (the longer a country has been a member, the higher the weight). The reasoning for this weighting system is based on the phenomenon that the longer a country has been in the E.U., the more time that country has had to better develop its social system and thus the more appealing they are likely to be to immigrants and migrants.

$$c_{E}=\sum_{i=1}^{27} p\left( d_{E}\left( i \right)-D_{E}\left( i \right) \right)^{2}$$

In order to minimize the distance between the value observed ($D_{E}$) and the value predicted $d_{E}$, the weights $w$ were optimized by searching for the smallest value defined as $c_{E}$ which corresponded to the best match between the U.S. states and the E.U. nations.

$$argmin_{w}(c_{E})$$

Then, we compared the percentile between our model and the observed data from official sources (Figure 2). The robustness of our model was self-evident as there was very little percentile difference between our model and the observed data.

The third step included projecting the infection rate of the U.S. by a dichotomized race/ethnicity category (white/Caucasian and minorities) to the E.U. state matching (Figure 3).

In the fourth step, the model projected the U.S. death rate per dichotomized race (white/Caucasian vs. minorities) to the E.U. state through matching (Figure 4).

Overall, these mathematical and statistical techniques bolstered the accuracy of the Covid-19 infection and death rates model to predict the Covid-19 infection and death rates per 100,000 residents projected by race (white Caucasian / minorities) onto the E.U. states.

A weight of 4 was allocated to Belgium, France, Germany, Italy, Netherlands Luxembourg (entered 01/01/1958), a weight of 3 was allocated to Denmark, Greece, Ireland, Portugal, Spain, (entered between 01/01/1973-01/01/1986) and a weight of 2 was allocated to Austria, Finland, Sweden (entered 01/01/195) and a weight of 1 was allocated to Bulgaria, Croatia, Cyprus, Czech Republic, Estonia, Hungary, Iceland, Latvia, Liechtenstein, Lithuania, Malta, Norway, Poland, Romania, Slovak, Republic, Slovenia (entered after 01/05/2004).
